# Midwives' use of best available evidence in practice: An integrative review

**DOI:** 10.1111/jocn.15027

**Published:** 2019-10-14

**Authors:** Annemarie De Leo, Sara Bayes, Sadie Geraghty, Janice Butt

**Affiliations:** ^1^ Edith Cowan University Perth WA Australia; ^2^ Charles Darwin University Darwin NT Australia; ^3^ King Edward Memorial Hospital Perth WA Australia

**Keywords:** evidence‐based practice, evidence‐to‐practice gap, maternity care, midwifery

## Abstract

**Aims and objectives:**

To synthesise international research that relates to midwives' use of best available evidence in practice settings and identify key issues relating to the translation of latest evidence into everyday maternity care.

**Background:**

Midwifery is a research‐informed profession. However, a gap persists in the translation of best available evidence into practice settings, compromising gold standard maternity care and delaying the translation of new knowledge into everyday practice.

**Design:**

A five‐step integrative review approach, based on a series of articles published by the Joanna Briggs Institute (JBI) for conducting systematic reviews, was used to facilitate development of a search strategy, selection criteria and quality appraisal process, and the extraction and synthesis of data to inform an integrative review.

**Methods:**

The databases CINAHL, MEDLINE, Web of Science, Implementation Science Journal and Scopus were searched for relevant articles. The screening and quality appraisal process complied with the PRISMA 2009 checklist. Narrative analysis was used to develop sub‐categories and dimensions from the data, which were then synthesised to form two major categories that together answer the review question.

**Results:**

The six articles reviewed report on midwives' use of best available evidence in Australia, the UK and Asia. Two major categories emerged that confirm that although midwifery values evidence‐based practice (EBP), evidence‐informed maternity care is not always employed in clinical settings. Additionally, closure of the evidence‐to‐practice gap in maternity care requires a multidimensional approach.

**Conclusion:**

Collaborative partnerships between midwives and researchers are necessary to initiate strategies that support midwives' efforts to facilitate the timely movement of best available evidence into practice.

**Relevance to clinical practice:**

Understanding midwives' use of best available evidence in practice will direct future efforts towards the development of mechanisms that facilitate the timely uptake of latest evidence by all maternity care providers working in clinical settings.

**What does this paper contribute to the wider global clinical community?**

- Understanding midwives use of best available evidence will direct future efforts to facilitate the practice of EBH in maternity services
- Evidence‐informed maternity care improves the standard maternity services and health outcomes of women and newborns
- Interdisciplinary collaboration between health organisations and practitioners will lead to improved uptake of latest evidence in practice settings

## 1 INTRODUCTION

Evidence‐based practice (EBP) is embraced internationally as the ideal approach to improving healthcare outcomes for consumers, using the best available evidence to inform policy and the practice of persons responsible for providing care (Miller et al., 2016). Within maternity services, EBP has been recognised as crucial for reducing the use of non‐evidence‐based information, which has been associated with the over‐medicalisation of normal pregnancy and birth (Miller et al., 2016). However, as research continues to provide clinicians with new evidence to inform practice, the timely uptake of best available evidence in clinical contexts remains inconsistent (Hines, Kynoch, Munday, & McArdle, 2017). This creates a considerable challenge for midwives, like other care providers, who are well aware of their obligation to practice evidence‐based care, but report difficulty implementing latest evidence into everyday practice (Bayes, Juggins, Whitehead, & De Leo, 2019; McVay, Stamatakis, Jacobs, Tabak, & Brownson, 2016).

Using best available evidence to inform policy and practice in midwifery is explicitly detailed in midwifery governance documents, for example the Australian Midwife Standards for Practice (NMBA, 2018). However, the pathway from evidence to practice is complex, and where latest evidence is recognised but not used in everyday care, a “gap” in translation has been conceptualised. The gap represents not only the delayed transfer of evidence into clinical contexts, but also the gap between knowledge producers and knowledge users (Rycroft‐Malone et al., 2016). A number of remedial approaches have been proposed to address this phenomenon in recent years, which are largely conceived from the fields of psychology and Implementation Science (IS) (Gagliardi, Berta, Kothari, Boyko, & Urquhart, 2016; Graham, Kothari, & McCutcheon, 2018; Tucker, 2017). However, there remains limited research on the use of evidence‐based information by midwives in maternity contexts.

### 1.1 Aims

The aims of this review were to present a synthesised summary of the findings from previous research that relates to midwives' use of best available evidence in practice settings and identify key issues relating to the phenomenon of interest.

## 2 METHODS

A systematic approach was used to facilitate development of a search strategy, selection and quality appraisal of studies. This was based on the Preferred Reporting Items for Systematic Reviews and Meta‐Analysis (PRISMA) checklist (Moher, Liberati, Tetziaff, & Altman, 2009; see Appendix S1) and a series of articles published by the Joanna Briggs Institute (JBI) outlining a step‐by‐step approach to conducting systematic reviews (Aromataris & Pearson, 2014; Aromstaris & Riitano, 2014; Munn, Tufanaru, & Aromataris, 2014; Porritt, Gomersall, & Lockwood, 2014; Robertson‐Malt, 2014; Stern, Jordan, & McArthur, 2014).

### 2.1 Search strategy

The purpose of this search strategy was to find published literature relevant to the topic of interest. This involved the formulation of a review question guided by the PICO criteria (“Population”, “Phenomenon of Interest” and “Context”) for qualitative studies. The question developed for this review was “Do midwives (P) always use best available evidence (I) in practice (Co)?*”* Selection criteria were established to determine which articles were eligible for review. This included original qualitative research and case studies, literature published between the years 2009–2019 and articles printed in English that were available in full text. Papers were excluded if they did not include midwifery participants, were not relevant to the review question or did not meet the selection criteria.

A search strategy was derived from keywords using a logic grid (Table 1) that were then combined to form a search string using the Boolean operators “AND” and “OR” (Table 2).

**Table 1:** Logic Grid: “Do midwives always use best available evidence in practice?”

Population (P)	Phenomenon of Interest (I)	Context (Co)
Midwi*	Evidence‐based practice	Practice setting
Nurse midwife*	Evidence‐based health care	“Maternity care”
“obstetric nurse”	EBP	Maternity unit
	EBH	Maternity setting
	“best practice”	“maternity care”
	“latest evidence”	Midwi* service*
	Evidence based health*	Clinical setting
		Hospital

**Table 2:** Search string derived from the Logic Grid

(Midwi*(tw) OR “nurse midwife” OR “obstetric nurse” AND latest evidence OR evidence based practice (tw) OR “EBP” OR evidence based OR best practice OR evidence based health* OR “EBH” OR “EBHC” AND midwi* care OR practice setting* OR “maternity care” OR “maternity unit” AND clinical setting OR practice setting OR “clinical practice” OR “point of care” OR midwi* service* OR midwi* unit OR hospital)

### 2.2 Quality appraisal

The process of study selection and quality appraisal was guided by the PRISMA framework (Moher et al., 2009) and included entering the search string into the electronic databases Cumulative Index to Nursing and Allied Health Literature (CINAHL), MEDLINE, Web of Science, Implementation Science Journal and Scopus. The selection criteria were then applied to focus the search on the review question and agreed criteria, which resulted in 133 papers (*n* = 133). These were screened by journal, title and abstract to establish the success of the search string and also eliminate irrelevant articles (*n* = 109). From this, a manual search for relevant publications by title and abstract in key midwifery journals (e.g. “Midwifery” and “Women and Birth”) was conducted, which retrieved four additional papers (*n* = 4). Twenty‐eight papers (*n* = 28) were retained for full‐text review based on relevance to the review question and adherence to selection criteria. This process was conducted by authors one and two (AD and SB).

### 2.3 Quality appraisal outcomes

**Figure 1:**
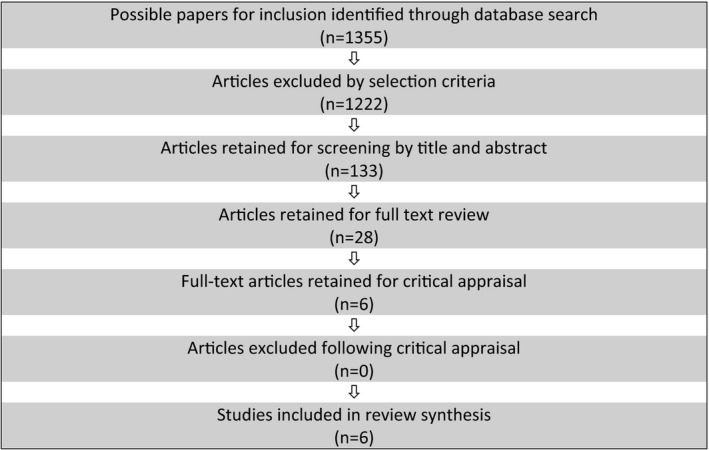
“Do midwives always use best available evidence in practice?”

A total of six papers (*n* = 6) were considered suitable for quality appraisal and were assessed against the JBI Critical Appraisal checklist for both cross‐sectional studies and qualitative research papers (Joanna Briggs Institute [JBI], 2014). This was conducted by all three authors independently. No papers were excluded following the appraisal process (*n* = 0). Therefore, a total of six studies (*n* = 6) were included in the review. These comprised of one qualitative research paper and five reporting cross‐sectional studies (Table 3). Collectively, the included papers report on midwives' use of best available evidence in Australia, the UK and Asia. The search and screening process is presented in Figure 1, adapted from the PRISMA flow chart for reporting the review process (Liberati et al., 2009).

**Table 3:** Synthesis of sub‐categories and major synthesised findings

Sub‐categories		Major synthesised findings
1. Midwifery values EBP and recognise non‐EBP is costly		1. Although midwifery values EBP and non‐EBP is costly, best available evidence is not always used in practice
2. Best available evidence is not always used in practice		
3. Factors preventing EBP are varied		
dimension 3.1: “there is no reason to change”		
dimension 3.2: “change is (too) hard”		
dimension 3.3: Time is an issue		
dimension 3.4: “Budget constraints are a limiting factor”		2. Factors preventing EBP are varied, and closure of the evidence–practice gap in maternity services requires a multidimensional approach
4. Closure of the evidence–practice gap in maternity care requires a multidimensional approach		
5. Attitudes towards EBP influence evidence‐based care		
6. Midwives do not have the confidence or skills to lead change implementation		

### 2.4 Data abstraction and synthesis

Data abstraction from the literature included in this review was guided by the approach described by Munn et al. (2014), where the findings of each study and their interpretation were extracted and then organised into sub‐categories and dimensions. These were then merged to form major categories agreed by all authors (AD, SB, SG and JB), which were used to synthesise information that represent what is known to date about midwives' use of best available evidence in practice.

## 3 RESULTS

An extensive search of the literature was conducted between January–March 2019, guided by the review question “Do midwives always use best available evidence in practice?” The initial search string (Table 2) retrieved 1,355 articles. The selection criteria were then applied, which excluded 1,222 papers. The resultant 133 papers were retained for screening by journal, title and abstract. Following this, 28 articles remained for detailed review of full text based on relevance to the review question and adherence to selection criteria. Six articles were selected for critical appraisal using the JBI Critical Appraisal checklist for both cross‐sectional studies and qualitative research papers (Joanna Briggs Institute [JBI], 2014), and quality appraisal was conducted by all authors independently. All papers scored 7 or above on the Critical Appraisal checklist (the highest possible score being 10), so all appraised articles were retained for inclusion in this review. A narrative summary of the papers included in this review is presented below.

### 3.1 Narrative summary of included studies

The first included paper, authored by Bayes et al. (2019), reported on the experiences of Australian change‐leader midwives' (*n* = 16) implementing evidence‐based innovations in midwifery practice settings. Using Glaserian grounded theory, the paper explored change‐helping or change‐hindering factors, which were compared to the seminal Consolidated Framework for Implementation Research (CFIR), a tool developed within the context of Implementation Science (IS) to identify environmental factors that influence the use and implementation of change initiatives. The study comprised change‐leader midwives who had tried to initiate a practice innovation in their workplace. Participants were interviewed via Skype or telephone by single in‐depth interviews guided by semi‐structured questions. Findings were analysed and developed into sub‐categories, which were then formed into major categories that described the phenomenon of interest.

The second paper included was a descriptive cross‐sectional study reporting Australian midwives' (*n* = 297) use of evidence‐based guidelines in clinical practice. Authored by Toolhill, Sidebotham, Gamble, Fenwick, and Creedy (2017), data were collected in a four‐sectioned survey. The first section collected demographic and personal information, and the second comprised a tool developed by the authors to determine midwives' perceptions of barriers to using best available evidence in practice. The third section asked respondents to use the Adaptive Evidenced‐Based Practice Beliefs (A‐EBP‐B) Scale to measure midwives' confidence to implement evidence in practice, and in the final section, participants were provided with a text box to make additional comments regarding the usefulness of normal birth guidelines. The findings indicated that although midwives considered they had sufficient knowledge and skills to practice evidence‐based care, they reported insufficient time, lack of collegial support and barriers from administrative processes to hamper their use of latest evidence in practice settings. Significantly, most participants reported feeling concerned that using latest evidence would result in midwives being blamed for adverse maternal or neonatal outcomes. Toolhill and team concluded that lack of organisational processes and a risk‐adverse culture hinder the use of evidence‐based guidelines and the uptake of latest evidence in practice settings.

The third paper by Veeramah (2016a) examined the use of evidence‐based information by nurses and midwives in a cross‐sectional online survey. The sample included nursing and midwifery diplomats and graduates (*n* = 172) from a single university in the UK and was conducted between June–December 2013. The web‐based software Qualitrix™ was used to develop the survey, which comprised of five sections: (a) participant professional profiles, (b) attitudes towards EBP, (c) use of latest evidence to inform clinical practice, (d) accessibility to resources and (e) skills to implement and EBP. The study found participants displayed positive attitudes towards the use of evidence‐based information in practice settings. However, factors hindering EBP were noted to include lack of resources (e.g. computer software and Internet access) to search for latest evidence and insufficient time to research during working hours. Additionally, perceived resistance from colleagues and managers was also considered to be a significant barrier to using latest evidence in practice. The author concluded that using evidence‐based information to inform practice is fundamental and providing time away from bedside responsibilities may improve midwives and nurses capacity to search and apply research findings to practice.

The fourth paper, authored by Fairbrother, Cashin, Conway, Symes, and Graham (2016), reported on a descriptive cross‐sectional study exploring the skill levels, behaviours and barriers of nurses and midwives in relation to EBP. Participants (*n* = 169) completed an online questionnaire comprising five domains: (a) practice knowledge bases, (b) barriers to finding and reviewing evidence, (c) barriers to changing practice, (d) support to implement change and (e) EBP skills. Descriptive analysis was conducted using spss software. The findings reported low levels of acceptance and understanding of evidence‐based knowledge, and inaccessibility to research material. Additionally, time‐related barriers were reported by participants to be a significant issue in preventing the uptake of latest evidence into practice settings. The authors concluded that knowledge not underpinned by best available evidence remains the most common basis for nursing and midwifery practice decisions. Recommendations for capacity building strategies such as research mentorships and ongoing education in evidence interpretation were considered valuable to improving both midwives and nurses confidence to incorporate best available evidence in clinical environments.

The fifth paper evaluated the provision of care provided to women (*n* = 24) during normal labour and childbirth across four public hospitals in Tehran. Pazandeh, Huss, Hirst, House, and Baghban (2015) investigated the quality of intrapartum care provided by midwives and other maternity staff, comparing clinical care to current EBP guidelines. Additionally, postpartum women (*n* = 100) were interviewed about their care during labour and childbirth prior to discharge. Findings described the use of non‐evidence‐based practices including routine augmentation, induction of labour and application of fundal pressure during the second stage of labour and routine episiotomy. Other clinical practices such as facilitating removal of the placenta during the third stage of labour, immediate skin‐to‐skin contact postpartum between mother and newborn, and early initiation of breastfeeding were observed to be aligned with latest evidence. Authors concluded that closing the evidence‐to‐practice gap remains challenging for maternity care providers. Recommendations for further research into strategies and solutions specific to care provider needs were suggested to facilitate the use of latest evidence in practice. Notably, opinion leaders and experienced midwives were emphasised to have a key role in changing care provider behaviour.

The final study by Heydari, Mazlom, Ranjbar, and Scurlock‐Evans (2014) reported on the evidence‐based knowledge, attitude and practice of nurses and midwives regarding clinical decision‐making and implementation of best available evidence. Set in Iran, a descriptive cross‐sectional study was conducted on nurses and midwives (*n* = 240), using two questionnaires, one collecting demographic information and the second examining participant's knowledge, skills, attitudes and use of EBP. spss software was used to analyse the data collected. Major findings identified that although most nurses and midwives express moderately positive attitudes towards EBP, sub‐optimal use of evidence‐based care in clinical settings was reported. Organisational culture was also influential to the attitudes and practice of EBP amongst participants, emphasising the need for EBP training and education in clinical settings. Recommendations were made to promote future collaboration between clinical practitioners, academic centres and researchers to improve the use of evidence‐based information in practice. Table 4 presents a summary of the papers retained for inclusion in this review.

**Table 4:** Papers retained for inclusion

Reviewed paper #	Year and journal	Title	Methods	Key findings
1. Bayes, S Juggins, E Whitehead, L De Leo, A	*Midwifery* (2019)	Australian Midwives' experiences of implementing practice change	Glaserian grounded theory	Obstacles at many levels impinge on the use of best available evidence in practice Ideal evidence‐based Practice is not always Reflected in day‐to‐day midwifery care
2. Toohill, J Sidebotham, M Gmble, J Fenwick, J Creedy, D	*Women and Birth* (2017)	Factors influencing midwives' use of an evidence based normal birth guideline	Descriptive cross‐sectional study	Organisational characteristics influence midwives' use of latest evidence in practice Implementation of EBP remains a challenge Interdisciplinary Collaboration is needed Improve the uptake of EBP
3. Veeramah, V	*Journal of Clinical Nursing* (2016)	The use of evidence‐based information by nurses and midwives to inform practice	Cross‐sectional online survey	Midwives have positive attitudes towards EBP EBP is not consistent in practice settings Care providers report difficulty interpreting the quality and technical language of research reports
4. Fairbrother, G Cashin, A Conway, R Symes, A Graham, I	*Collegian* (2016)	Evidence based nursing and midwifery practice in a regional Australian healthcare setting: Behaviours, skills and barriers	Cross‐sectional descriptive survey	Midwives lack the confidence to translate latest evidence into practice Workplace culture is not always receptive to change Interdisciplinary support Would facilitate the practice of evidence‐informed care
5. Pazandeh, F Huss, R Hirst, J House, A Baghban, A	*Midwifery* (2015)	An evaluation of the quality of care for women with low risk pregnancy: The use evidence‐based practice during labour and childbirth in four public hospitals in Tehran	Descriptive evaluation study	Closing the evidence‐practice gap demands a multidimensional approach The cost of non‐EBP is considerable and difficult to justify Midwives can be the change leaders of EBP
6. Heydari, A. Mazlom, S Ranijbar, H Scurlock‐Evans, L	*Worldviews on Evidence‐based Learning* (2014)	A study of Iranian Nurses' and midwives' knowledge, attitudes and implementation Evidence‐Based Practice: the time for change has arrived	Descriptive cross‐sectional study	Midwives require resources and organisational support to lead change initiatives There is need to promote a climate of change in healthcare organisations

### 3.2 Findings

Sixty findings and interpretive statements were extracted from the six articles selected for inclusion in this review. From these, six sub‐categories emerged that were then collapsed into two major synthesised categories. One sub‐category, number four, is four‐dimensional. Collectively, the findings confirm that using best available evidence in practice is challenging for midwives and is subsequently not always applied to everyday maternity care. This demonstrates the evidence‐to‐practice gap persists in midwifery and that resolution of this issue requires interdisciplinary collaboration and timely actions.

#### 3.2.1 Major synthesised category 1: Although midwifery values EBP and non‐EBP is costly, best available evidence is not always used

This synthesised finding was developed from two sub‐categories (numbers one and two) that emerged from 19 findings and reflects the sub‐optimal use of best available evidence in practice, despite midwives' value of EBP and the costly outcomes of non‐evidence‐based care on maternity services. For the majority of participants, the philosophy of midwifery care aligns with promoting EBP; however, midwives recognise that using latest evidence in practice is sub‐optimal, which can result in midwifery care that is harmful to the well‐being of women and neonates, and difficult to justify.

#### 3.2.2 Major synthesised category 2: Factors preventing EBP are varied, and closure of the evidence–practice gap in maternity care requires a multidimensional approach

Four categories, which were derived from four sub‐categories (numbers three, four, five and six) and 41 findings, merged to develop this synthesised finding. While various factors limit midwives efforts to use best available evidence in practice, organisational characteristics such as workplace culture, interdisciplinary collaboration and attitudes towards EBP have been recognised as crucial drivers of change. A multidimensional approach is needed to resolve the existing evidence‐to‐practice gap in maternity care, with midwives' key stakeholders in closing the gap and changing care provider behaviours.

#### 3.2.3 Sub‐category 1: Midwifery values EBP and recognise non‐EBP is costly

This category conveys midwifery's value of EBP and the cost of non‐EBP to both women and health organisations. Pazandeh et al. (2015) established that “midwifery aims for [an] evidence based model of care and promotes EBP”, which was consistent with author's findings in paper two, who suggested “midwives philosophies align with [EBP] guidelines (Toolhill et al., 2017, p. 121)”. In paper three, Veeramah (2016a, p. 346) confirmed EBP to be a valuable element of midwifery care and important in the “daily practice of nurses and midwives”. The issue of non‐evidentiary‐based care was discussed by Toolhill et al. (2017), who stated “the cost of [unjustified] interventions are considerable and difficult to justify”. Similarly, midwives reported unnecessary or ineffective care led to “some practices do[ing] more harm than good” (Toolhill et al., 2017, p. 417).

#### 3.2.4 Sub‐category 2: Best available evidence is not always used in practice

These data describe midwives' use of best available evidence in practice. It emerged that midwifery care is not always reflective of EBP, nor used routinely in everyday care of patients (Bayes et al., 2019). It was reported that national (EBP) guidelines were not followed consistently in maternity settings (Pazandeh et al., 2015), which confirmed comments by midwives in paper two, who were aware of (EBP) guidelines, but indicated they were not always used to inform clinical practice (Toolhill et al., 2017). Observational examples of non‐EBP were reported by Panzandeh and team, such as the use of fundal pressure and routine episiotomy during the second stage of labour. Additionally, authors observed the “the use of partograms [to be] irregular…or filled in after delivery” and “induction of labour was observed as routine practice” (Pazandeh et al., 2015, p. 1050). Other midwives acknowledged that correct use of evidence‐based practices was sometimes ignored, which resulted in higher rates of intervention during labour. For example, “the majority of women's labour was checked like high risk women” and “women admitted in early labour were routinely augmented despite being a low risk pregnancy” (Pazandeh et al., 2015, p. 1050).

#### 3.2.5 Sub‐category 3: Factors preventing EBP are varied

This category explored the various factors that hinder midwives' efforts to adopt EBP in clinical contexts. It was identified that organisational characteristics and workplace culture were influential to midwives' use of evidence‐based information in practice (Toolhill et al., 2017). Additionally, authors Bayes et al. (2019) suggested midwives were obstructed at many levels, which impinged on their ability to use latest evidence in practice. Common barriers to EBP resonated across all papers and included unsupportive workplaces, collegial resistance to change, insufficient time and budget constraints. These were considered additional dimensions of sub‐category three and are detailed below.

#### 3.2.6 Dimension 3.1: there is no reason to change

Unsupportive colleagues were reported as a significant obstacle to using latest evidence in the workplace, as exemplified by one midwife who recalled a colleague's resistance to implementing a new practice; “why should we change something that we've been doing for twelve years?” (Bayes et al., 2019). This feeling was not uncommon, with Veeramah also reporting that midwives recognised their reluctance to change despite new innovations being introduced, preferring to work “the way we have always done it” because it has “worked for us for years” (Veeramah, 2016a, p. 348).

#### 3.2.7 Dimension 3.2: change is (too) hard

Resistance to change was identified by midwives as another barrier to using evidence‐based information. This was explained by midwives in paper two, who described EBP as “difficult” and “challenging” (Toolhill et al., 2017, p. 420). It was further confirmed by midwifery change leaders, who discussed the opposition they received when trying to implement latest evidence into practice, with comments such as “change has never been embraced [in my workplace]” and “the resistance to change was phenomenal”, illustrating the hardship midwives faced when trying to lead change initiatives (Bayes et al., 2019, p. 40).

#### 3.2.8 Dimension 3.3: Time is an issue

Insufficient time was reported to obstruct midwives efforts to use of latest evidence in everyday maternity care. One midwife suggested “EBP takes too much time” (Toolhill et al., 2017, p. 421), while others reported “we can't do it because we're too busy doing the day‐to‐day production line of work” (Bayes et al., 2019). Other midwives described finding time to source latest evidence during work hours near impossible, as one midwife explained “I don't have time to locate evidence‐based information at work” (Fairbrother et al., 2016, p. 32).

#### 3.2.9 Dimension 3.4: Budget constraints are a limiting factor

Budget constraints were considered a limiting factor for midwives trying to implement change initiatives that promoted EBP. One midwife declared her efforts to implement a practice change were hampered by her workplace, who “wouldn't support evidence‐based initiatives unless they were resource‐neutral” (Bayes et al., 2019, p. 42). Another limiting factor was inadequate funding for “computers with internet services in suitable work spaces” (Toolhill et al., 2017, p. 421), which compromised midwives efforts to access literature and implement evidence‐based information into clinical care.

#### 3.2.10 Sub‐category 4: Closure of the evidence–practice gap in maternity care requires a multidimensional approach

Closing the evidence–practice gap in maternity care requires collaboration and action between the varied disciplines of maternity care, as findings in this category articulate. Authors Toolhill et al. (2017, p. 421) recommended “interdisciplinary collegial dialogue around implementing best practice” to be essential in promoting the successful implementation of EBP. This resonated with Heydari et al. (2014, p. 329) who recommended researchers try “to work alongside practitioners to better understand the evidence‐base needed to support clinical practice”. The norms and values of an organisation were also recognised as “important drivers of practice…and change” (Fairbrother et al., 2016), while paper six suggested interdisciplinary collaboration between maternity care providers was a “crucial component of facilitating the use of evidence‐based healthcare” (Heydari et al., 2014, p. 330). Significantly, what resonated was the need to promote a climate of change (Heydari et al., 2014), and experienced midwives were considered key leaders in changing care provider behaviours (Pazandeh et al., 2015).

#### 3.2.11 Sub‐category 5: Attitudes towards EBP influence evidence‐based care

In this category, midwives' attitudes towards EBP and their practice of evidence‐based care are described. It was reported midwives have “moderately positive” attitudes towards EBP (Pazandeh et al., 2015). Similarly, Veeramah (2016a) suggested midwives who displayed positive attitudes towards EBP were more likely to use evidence‐based information to inform their clinical practice. The authors Heydari et al. (2014) identified a correlation between positive attitudes towards EBP and the successful adoption of latest evidence by care providers. This resonated with midwives who acknowledged that team culture was a significant influence in the uptake of latest evidence in clinical areas (Fairbrother et al., 2016). On the contrary, Bayes et al described the frustration midwives experienced from colleagues, administration and management, who expressed negative attitudes towards the use of EBP (Bayes et al., 2019). Similarly, midwives described the significant medical opposition and negativity from administration and colleagues towards practice change, as one midwife suggested “latest evidence is not always endorsed by management” (Bayes et al., 2019, p. 40).

#### 3.2.12 Sub‐category 6: Midwives do not have the confidence or skills to lead change implementation

Findings in this category confirm midwives lack confidence and the skills to lead change initiatives. Midwives reported feeling unsure of how to put evidence into practice, and lacked confidence in judging the quality and implications of research findings in their own practice (Toolhill et al., 2017). This was exemplified by Fairbrother and team, as one midwife voiced “difficulty interpreting statistical information and the technical language of research” (Fairbrother et al., 2016, p. 32). This resonated with statements made by midwives who claimed research reports were “not [made] readily available” or were “too difficult to understand” (Veeramah, 2016a, p. 344). These factors compromised midwives' efforts to lead change initiatives to employ EBP.

Collectively, the findings and their interpretation from the six articles included in this review describe midwives' use of best available evidence in practice. Notably, only two papers reported exclusively on midwives experiences of using best available evidence in practice (Bayes et al., 2019; Toolhill et al., 2017), the remaining four papers reported on a range of maternity care providers (e.g. obstetric nurses, midwives and obstetricians), although did not specify the sample size of each discipline. This limited the authors' ability to establish the absolute number of midwives comprising this review. However, it may be reasonably assumed that findings adequately reflect the midwifery profession's use of best available evidence in practice.

## 4 DISCUSSION

This review provides a synthesis of the existing literature relating to midwives' use of best available evidence in practice. The literature search and screening process resulted in six articles being assessed as suitable for inclusion. Following analysis, six sub‐categories were developed, which were merged to form two synthesised findings. The findings together characterise the attitudes and values of midwives towards EBP, and their use of evidence‐based information in clinical practice. Also identified are the various factors that impinge on midwives' use of best available evidence, resulting at times in sub‐optimal care and costly outcomes for women, newborns and health services. Although a systematic approach to the search and screening process was conducted, there is always a risk that pertinent studies relevant to the review question have been missed for inclusion. Authors of this review included studies only written in the English language, which may have excluded articles relevant to the topic. However, the six studies identified represent an international cohort of midwives and other maternity care providers from a range of maternity care settings. Therefore, the authors are cautiously confident this review provides an appropriate representation of midwives' use of best available evidence in practice.

The first major synthesised category “Although midwifery values EBP and non‐EBP is costly, best available evidence is not always used”, confirms that although best available evidence is not always used in practice, midwives value the philosophy of EBP and have a crucial role in facilitating the implementation of evidence‐based maternity care. Similarly, the principles of EBP are broadly accepted across a range of healthcare providers, including “physicians, nurses, pharmacists and dentists” (Mariano, Souza, Cavaco, & Lopes, 2018, p. 1), although remain underused in practice.

The expectation that new knowledge will translate into everyday practice is commonly misjudged, as care based on tradition or clinical experience, rather than best available evidence, continues to inform the practice of some healthcare providers (Graham et al., 2018; Nagpal, Sachdeva, Sengupta, Bhargava, & Bhartia, 2015). In Australia, mandatory regulations for midwives explicitly state that clinical practice must be informed by high‐quality evidence (NMBA, 2018). However, midwives like other care providers often find this difficult to achieve (Veeramah, 2016b).

Research investigating the sub‐optimal use of evidence in practice has produced a range of theories and resources from the field of Implementation Science (IS), an area of scientific study promoting the systematic uptake of best available evidence into healthcare practice (Nilsen, 2015). Seminal work in IS has led to expanding interest in knowledge translation and the gap between evidence‐to‐practice in health care (Casey, O' Leary, & Coghlan, 2018). To facilitate this process, a range of theories and frameworks have been developed to guide the dissemination–implementation process and inform clinicians of the actions needed to expedite the process (Casey, O' Leary, & Coghlan, 2018; Rycroft‐Malone, 2016). Arguably, one of the founding IS instruments is The Consolidated Framework for Implementation Research (CFIR), which was developed to promote implementation theories and define “what works where and why” (Damschroder et al., 2009, p. 1). Consisting of five domains (intervention characteristics, outer setting, inner setting, characteristics of individuals and processes), the framework highlights the barriers and facilitators of the implementation process, provides an implementation pathway and gives meaning to implementation outcomes (Keith, Crosson, O'Malley, Cromp, & Taylor, 2017). The CFIR, along with other IS theories and frameworks, has been considered useful by the nursing profession, although remains underused in midwifery contexts (Bayes, Fenwick, & Jennings, 2016). Breimaier, Heckemann, Halfens, and Lohrmann (2015) assert the frameworks to be too generic, needing adaptions to improve the usability and value of such tools in clinical contexts. Suggestion for the use of IS tools in midwifery has been considered a pathway to improving the uptake and use of evidence in practice, however, as yet existing tools have not significantly contributed to improving the use of best available evidence in practice (Seers et al., 2018). Further research is needed to ensure midwives are confident and adequately supported to lead change initiatives that promote EBP.

The second synthesised major category “Factors preventing EBP are varied, and closure of the evidence–practice gap in maternity care requires a multidimensional approach”*,* highlights the challenges of initiating EBP changes and the interdisciplinary approach needed to optimise the use of best available evidence in maternity services. Factors preventing the uptake of EBP are well documented in the literature and often prevent the adoption of best practice by clinicians (Colquhoun, Squires, Kolehmainen, Fraser, & Grimshaw, 2017). In the past, midwives amongst other care providers have identified these factors as “barriers”, such as workplace culture, time constraints, funding and resources and resistance to change (Barwick, 2011; Kennedy, Doig, Hackley, Leslie, & Tillman, 2012). These barriers impinge on clinicians' efforts to adopt new practice or process initiatives (Bayes et al., 2016; Darling, 2016; Geerligs, Rankin, Shepherd, & Butow, 2018; Weir, Newham, Dunlop, & Bennie, 2019). More recently, recognition of other dimensions influential to the implementation process is reported to include individual mindset, knowledge and values of EBP, clinical competence, confidence and collegial collaboration (Mariano et al., 2018). A study by Colquhoun et al. (2017) established a relationship between the uptake of EBP and four principle variables, competence and professionalism, perceived knowledge of research, perceived knowledge of EBP and access to information databases. All relate to the perceived values of EBP by clinicians, and their confidence and competence to implement best available evidence in workplace environments. Notably, authors emphasised the value of managerial and inter‐professional collaboration to optimise implementation outcomes.

This review identified organisational and interdisciplinary co‐operation to be crucial components of initiating the implementation and use of best available evidence by midwives. As illustrated by Hespe, Rychetnik, Peiris, & Harris (2018), organisational co‐operation was investigated using a team‐based approach to improve the uptake of evidence‐based guidelines in three Australian primary healthcare services. Interdisciplinary teams were developed to target specific practice improvements, which saw support from clinicians who identified “working as a team with shared responsibilities” a valuable component of implementing quality improvement (QI) initiatives across all disciplines of health care (Hespe et al., 2018, p. 5).

In maternity contexts, midwives are considered key stakeholders in the regulation of EBP initiatives (Renfrew et al., 2014); however, findings of this review assert that midwives continue to exhibit low levels of confidence and skills in interpreting and translating evidence‐based information into clinical practice. This issue is well recognised and documented within nursing literature, as illustrated by Mallion and Brooke (2016, p. 152), who report that for many nurses “lack of knowledge and skills of EBP remain[s] a major concern”. Prominent evidence implementation academic Rycroft‐Malone proposes that use of latest evidence in practice contexts is shifting towards a more socially constructed view, where “collaboration, partnership and engagement” between relevant stakeholders (clinicians, managers and policy makers) could see improvements to the uptake and use of evidence in everyday practice (Rycroft‐Malone et al., 2016, p. 221). Arguably, incorporating a multidimensional approach to the evidence‐to‐practice gap in midwifery could see the development of a resource designed specifically for midwifery contexts to support their use of best available evidence in maternity care services.

## 5 CONCLUSION

The consensus, both nationally and internationally, is that using best available evidence in practice is a priority issue for midwives and other maternity care providers. If the uptake of latest research findings continues to flounder, optimal health outcomes for women and newborns cannot be assured. However, supporting midwives with time away from the bedside, a workplace supportive of EBP and resources to facilitate their efforts may see the provision of evidence‐based maternity care become a reality. To close the persistent evidence‐to‐practice gap in maternity care, interdisciplinary collaboration and action between health organisations, midwives and researchers are recommended.

## 6 RELEVANCE TO CLINICAL PRACTICE

The pathway towards evidence‐based maternity care is inextricably linked to the emergence of new and innovative evidence. This review highlights that despite ongoing development and dissemination of high‐quality evidence, the translation of latest evidence into clinical practice remains sub‐optimal. Despite positive attitudes by midwives and other maternity care providers towards the use of best available evidence, concern regarding insufficient time, administrative barriers and lack of collegial support influence their capacity to implement EBP in clinical settings. The evidence‐to‐practice gap in maternity services remains a global issue for midwives and demands prompt action from both knowledge producers and knowledge users. Investing in strategies that support collaboration between midwives, researchers and maternity services could see the development of a resource designed by midwifery change leaders to bridge the gap from evidence‐to‐practice in maternity services.

## CONFLICT OF INTEREST

The authors declare no conflict of interest.

## AUTHOR CONTRIBUTIONS

Study design, literature search and screening process, quality appraisal and manuscript preparation: A. De Leo; literature analysis and manuscript preparation: S. Bayes; study design, quality appraisal and editing: S. Geraghty; quality appraisal and editing: J. Butt.
